# Apocynum Cannabinum in Anasarca

**Published:** 1881-06

**Authors:** 


### APOCYNUM CANNABINUM IN ANASARCA.

Bright's disease is becoming the fashionable disease to study, more especially since Charcot, who sets the fashion for many physicians in the United States, has been paying much attention to it; these symptoms have been chiefly pathological and symptomatological. However, many independent observers have dealt with it from the therapeutical aspect, and Dr. J. S. Dabney (*New Orleans Medical and Surgical Journal*, February, 1881,) has found, he claims, that apocynum cannabinum is one of the best diuretics and hydrogogue cathartics that can be employed in the disease, as it causes not only marked diminution of the anasarca, but also decrease in the albumen and casts. He claims for it certain advantages: First, a small quantity only is necessary to produce diuresis, emesis or catharsis. Second, it has an agreeable aromatic taste. Third, it has tonic properties. Fourth, its harmlessness; free emesis resulting from an overdose. While many of these claims seem rather strained, still there appears to be but little doubt that the remedy is of much value in ascites, anasarca and allied conditions.—*Medical Review.*

